# An assessment of the influence of elevated hygiene hazards and quality management systems on the safety of laminated and unlaminated films employed in the food sector

**DOI:** 10.1007/s10068-025-01926-8

**Published:** 2025-07-24

**Authors:** Romina Alina Marc, Crina Carmen Mureşan, Alina Narcisa Postolache, Florina Stoica, Ioana Cristina Crivei, Ionuţ-Dumitru Veleşcu, Roxana Nicoleta Raţu

**Affiliations:** 1 Food Engineering Department, Faculty of Food Science and Technology, University of Agricultural Science and Veterinary Medicine Cluj-Napoca, 3-5 Calea Mănăştur Street, 400372 Cluj-Napoca-Napoca, Romania; 2 GFSI (IFS, FSSC22000) International Lead Auditor & Consultant, Bucharest, Romania; 3 Department of Pedotechnics, Faculty of Agriculture, “Ion Ionescu de La Brad, Iasi University of Life Sciences, 3 Mihail Sadoveanu Alley, 700489 Iasi, Romania; 4 Department of Food Technology, Faculty of Agriculture, ”Ion Ionescu de La Brad, Iasi University of Life Sciences, 3 Mihail Sadoveanu Alley, 700490 Iasi, Romania

**Keywords:** PRPs, Hygiene, Risk, HACCP, Packaging

## Abstract

Packaging can be a major source of food contamination. To ensure the safety of the final packaging for the food industry, it is imperative to undertake a thorough assessment of potential contaminants that may come into contact with the food. This examination begins with evaluating the materials used in the production process of the packaging. To manufacture these packages for the food sector, the production units must comply with the first operating programs, develop a HACCP plan that conforms to the revised codex standards (which will take effect in 2023), and adopt IFS PACsecure (version 2, planned for 2023). The objective of this study is to assess the risks to the safety of laminated and unlaminated films utilized in the food business, in accordance with existing regulatory regulations and IFS Food Packing safety standards, within a food industry packaging manufacturing facility.

**Supplementary Information:**

The online version contains supplementary material available at 10.1007/s10068-025-01926-8.

## Introduction

The purpose of food packaging is to preserve the quality of food from production to its consumption. Different products have different demands. Certain products are susceptible to light or microbe development, while others are sensitive to moisture or gen. As a result, there is no universal packaging option; instead, each product's specific packaging needs to be decided upon. The most frequent quality losses are related to the transmission of oxygen and water vapor (Tihminlioglu et al., 2010).

The primary objective of packaging systems is to maintain the desired sensory attributes of the food while ensuring its microbiological safety, convenient handling, and provision of relevant information to the consumer. The package must serve as a barrier against oxygen, moisture, aroma, or light, depending on the sensitivity of the food and the environmental circumstances, to maintain the sensory characteristics of the meal being ingested (Tyagi et al., 2021; Usturoi et al., 2017).

The term packaging refers to the whole of the materials used to protect and hold a packaged good (Hong et al., 2021). The main objective of packing materials is to safeguard packaged items from aromas, dust, shocks, germs, humidity, light, temperature fluctuations, physical harm, breakages, and vibrations during storage or delivery, all of which have the potential to undermine their quality or safety. Additionally, it seeks to reduce the amount of food that is lost or wasted (Alamri et al., 2021; Han et al., 2018).

Plastics are the most common packaging material used by different food industries (Cruz et al., 2019 ) followed by paper and cardboard, glass, metallic materials, wood, and laminates (Alamri et al., 2021). A wide range of plastics are utilized in the food industry, such as polyethylene terephthalate (PET), polypropylene (PP), polystyrene (PS), polycarbonate (PC), polyvinylidene chloride (PVDC), high-density polyethylene (HDPE), low-density polyethylene (LDPE), medium-density polyethylene (MDPE), and multilayer materials, which are formed by amalgamating various types of plastics (Cruz R. M. S., 2019). According to Hang et al. (2021), the key attributes of packaging materials encompass barrier properties (such as resistance to gases, water vapor, and aromas), antimicrobial functionalities, mechanical features (including strength and tensile properties), optical properties, thermal properties, and environmental sustainability. Plastics possess various advantages, such as affordability, malleability, durability, lack of odor, water resistance, lightweightness, and low energy use during production (North and Halden, 2013; Wu et al., 2017). However, these materials pose a safety risk when they are in contact with food due to the presence of various chemical compounds. These compounds, such as additives, residues from raw materials, newly formed molecules, by-products of synthesis processes, antioxidants, plasticizers, monomers, and oligomers, can contaminate the packaged product during processing or packaging (Alamri et al., 2021; Wu et al., 2017). Emerging packaging technologies, such as active and intelligent packaging, are being developed to enhance food preservation and reduce waste. Active packaging incorporates substances that can absorb or release compounds (e.g., oxygen scavengers, antimicrobial agents), while intelligent packaging uses indicators to monitor product freshness or temperature abuse (Suppakul et al., 2003; Rastogi and Samyn, 2015). These innovations aim to improve food safety, extend shelf life, and support sustainable packaging strategies.

Using recycled bioplastics and biodegradable materials for food packaging is more advantageous than using single-use plastics. Bioplastics provide various innovative packaging methods such as controlled release (the gradual release of active ingredients), barrier technologies (preventing the passage of gases and liquids), scavenging (absorbing unwanted substances), as well as biodegradability and non-toxicity. To improve the functional and protective properties of food packaging, active compounds obtained from both organic and inorganic materials can be incorporated using coating, lamination, and dispersion techniques (Rastogi and Samyn, 2015).

Conventional processes are inadequate in managing the initial process risks to guarantee the hygiene and integrity of the end goods (Allata et al., 2017). Adhering to the principles of Hazard Analysis of Critical Control Points (HACCP), Good Manufacturing Practices (GMP), and Good Hygiene Practices (GHP) is crucial for maintaining food safety. All small and medium-sized food firms operating in the European Union (EU) are required to implement the Hazard Analysis and Critical Control Points (HACCP) system. The standard is internationally acknowledged for mitigating the hazards linked to foodborne diseases (Panghal et al., 2018). The ISO 22000:2018 standard integrates the concepts outlined in the Codex Alimentarius recommendations (22000:2018, 2018). The deployment of HACCP systems does not inherently lead to the creation of a traceability system, despite the usage of documentation methods. However, the execution of such a system is of utmost significance. Although Principle 7 of the HACCP system mandates meticulous documentation and record-keeping procedures, the implementation of traceability solutions is discretionary (Chhikara et al., 2018).

The IFS Packaging Guideline provides support in implementing the criteria of the IFS Food Standard. The guideline primarily focuses on food product providers and aims to enhance coordination across the supply chain to ensure the provision of safe products. ISO 22000 mandates that companies perform risk studies to identify significant dangers. Additionally, it adheres to the core tenet of both ISO 22000:2018 and Codex HACCP, which mandates the performance of hazard analysis. The objectives of HACCP systems are to identify, evaluate, and manage hazards. The Global Food Safety Initiative (GFSI) has rejected ISO 22000 as a standard reference for food manufacturers because it lacks relevant PRP (prerequisite program) data. ISO22000:2018 introduces modifications that revolve around the implementation of risk-based thinking and risk reduction. The main focus is on the identification of a PRP (pre-requisite program) and a CCP (critical control point) for significant risks (Chen et al., 2019; Chhikara et al., 2018; ISO^2200^0:^2018^; Panghal et al., 2018). The Global Food Safety Initiative (GFSI) has acknowledged the FSSC 22000 and the IFS Foods as meeting its standards. FSSC 22000, in accordance with ISO 22000:2018, mandates the inclusion of Part II 2.1.4 (March 2020) as a supplementary prerequisite. Requirement: ISO/TS 22002–1:2009 is necessary before proceeding. Due to their shared objectives, the standards possess similar criteria and a certain level of correlation. The audit level, however, varies greatly as it utilizes multiple levels, system points, and categories (Chen et al., 2019).

In this context, there is an increasing necessity to ensure the safety of packaging materials used in the food industry, especially regarding potential risks related to chemical migration and microbiological contamination.

Therefore, the present study aims to assess the safety and security hazards associated with laminated and unlaminated films used in the food industry.

The focus is on identifying key risk factors and control measures, in alignment with IFS Food Packaging safety standards and statutory requirements.

This study is significant because it addresses industry challenges concerning food-grade packaging compliance, consumer health protection, and alignment with updated international regulations in packaging production environments.

## Materials and methods

### Sample collection

The study assessed 21 food contact materials utilized for packaging a range of foods including cereal and cereal products, chocolate and confectionery, fruits, vegetables and their derivatives, fats and oils, dairy products, and several other products. The products were sourced from a Romanian food packaging manufacturer and were stored under controlled laboratory conditions prior to analysis.

The samples included a variety of foils and films, such as polyethylene foil (PE), polypropylene foil (PP), polyethylene terephthalate foil (PET), ethylene copolymer and vinyl alcohol foil (EVOH), polyamide foil (PA), biaxially oriented polypropylene foil (BOPP), cast polypropylene film (CPP), metalized biaxially oriented polypropylene (BOPP metalized), metalized cast polypropylene (CPP metalized), aluminum foil (AL), metalized polyethylene terephthalate (PET metalized), and paper foil (PAP). These materials can be found in various combinations on the market, such as laminated films used for automated packaging (e.g., CPP/PET), triplex films (e.g., PE/PAP/AL), and films designed for automatic packaging (e.g., PE/EVOH/PE).

### Assessing the food risk assessment scheme in food packaging production facilities to the established food safety initiatives

The risk assessment approaches applied in food packaging production facilities include Good Hygiene Practice (GHP), Good Veterinary Practice (GVP), Good Distribution Practice (GDP), and Good Commercial Practice (GTP). These were evaluated through documentation review, process observation, and personnel interviews, focusing on critical control implementation. The standards mentioned are Hazard Analysis and Critical Control Points (HACCP), ISO 22000, IFS Food v8 2023, FSSC 22000 v6 April 2023, and GFSI-recognized standards.

### Elaboration of PRPs

The performance of PRPs (GMPs, GHPs, GVPs, GTPs, SSOPs) was carried out according to the methods given by Cusato (Cusato et al., 2013) and in compliance with the provisions of the Codex Alimentarius of 2023 (WHO, 2023).

PRPs were identified and verified through checklists and physical inspections of facility layout, sanitation protocols, equipment condition, and employee hygiene practices.

### Elaboration of the HACCP Plan

The HACCP plan was developed using the framework established by Muresan et al. (Mureşan et al., 2020), but it was modified to meet the specifications of the updated edition of Codex Alimentarius 2023 (WHO., 2023), FSSC 22000 v6 (April 2023) (V6, 2023), and IFS Food v8 (April 2023) (v8, 2023).

The process included hazard identification, risk assessment using a severity-probability matrix, and the definition of CCPs and corresponding monitoring and corrective actions.

### TACCP analysis

The TACCP (Threat Assessment Critical Control Point) analysis regarding the prevention of malicious threats to food, such as sabotage, extortion or terrorism were carried out according to the requirements of IFS Food (v8, 2023) and IFS PACsecure (IFS PACsecure Standard (version 2, 2023).

The analysis was performed through structured vulnerability assessments, access control mapping, and threat scenario simulations, focusing on intentional adulteration and insider threats.

### Sample preparation

The global migration of components was tested using the gravimetric method specified in the EN 1186:2003 standard, namely parts 2, 7 and 13 (Plastics, 2002). This method assesses all the chemical components that have the potential to migrate from the packaging being examined into the food product.

Samples were cut into standard-sized test specimens, conditioned under controlled humidity and temperature, and exposed to appropriate simulants in sealed vessels.

Following this criterion, the process of preparing plastic samples for extraction was executed. The test conditions employed in this investigation adhered to the specifications outlined in Regulation (EU) No 10/2011 (10/2011, 2011), the experiment involved conducting tests on the samples for a duration of 10 days, specifically at a temperature of 40°C. This method is widely recognized as the most commonly employed approach (Paseiro-Cerrato et al., 2019). According to Ardic (Ardıç, et al., 2015), the evaluation of the migration of chemical components from food packaging to food simulants involves two separate phases. Firstly, the polymer packaging should be exposed to the food simulant in order to enable the transfer of chemicals from the packaging material into the simulant. The second purpose is to quantify the aggregate number of migrants that are conveyed to a food simulant, either in terms of overall migration (OM) or specific migration (SM) (Schmid and Welle, 2020).

The evaluation of the worldwide movement of the components was carried out using food simulants: simulant A—10% ethyl alcohol, simulant B—3% acetic acid, simulant D2—olive oil, and simulant E—tenax. Following the completion of the extraction phase, the obtained extract was evaporated in a water bath utilizing platinum crucibles. The residue was weighed, and the final quantification was expressed in milligrams per square decimeter (mg/dm^2^), ensuring consistency across all test materials.

### Microbiological analysis

The approach developed by Ruiz-Llacsahuanga was used to conduct microbiological control for coliform bacteria (Ruiz-Llacsahuanga et al., 2021) The determination of the overall bacterial count and presence of molds was conducted using the methodology outlined by Suppakul (Suppakul et al., 2003).

Sterile swabs were used to collect surface samples from each material under aseptic conditions, followed by cultivation on selective media. Incubation was performed under standard conditions (e.g., 37 °C for bacteria, 25 °C for molds) and CFU/cm2 were recorded.

### Results interpretation

The experiment was conducted three times, and the results were presented as the average values with the corresponding standard deviation.

Data normality and variance homogeneity were verified before performing statistical tests.

The Tukey's test, in conjunction with one-way analysis of variance (ANOVA), was utilized to evaluate the differences among samples, assuming equal variances and a normal distribution of data. Significance was determined at a confidence level of 95% (p < 0.05).

## Results and discussion

### Evaluating hazards and establishing tolerable thresholds

The study analyzed 21 types of foils and films, including polyethylene (PE), poly-propylene foil (PP), polyethylene terephthalate (PET) foil, ethylene copolymer and vinyl alcohol foil (EVOH), polyamide foil (PA), biaxially oriented polypropylene film (BOPP), cast polypropylene film (CPP), biaxially oriented metallized polypropylene (metallized BOPP), metallized cast polypropylene (metallized CPP), aluminum foil (AL), metallized polyethylene terephthalate (metallized PET), and paper film (PAP). On the market, these materials are available in several types of combinations as laminated films for automatic packaging (e.g. CPP/PET), triplex films (e.g. PE/PAP/AL) and automatic packaging films (e.g. PE /EVOH/PE). These foils were made following all the stages of the flow chart described in Supplementary Materials Fig. 1.

**Fig. 1:**
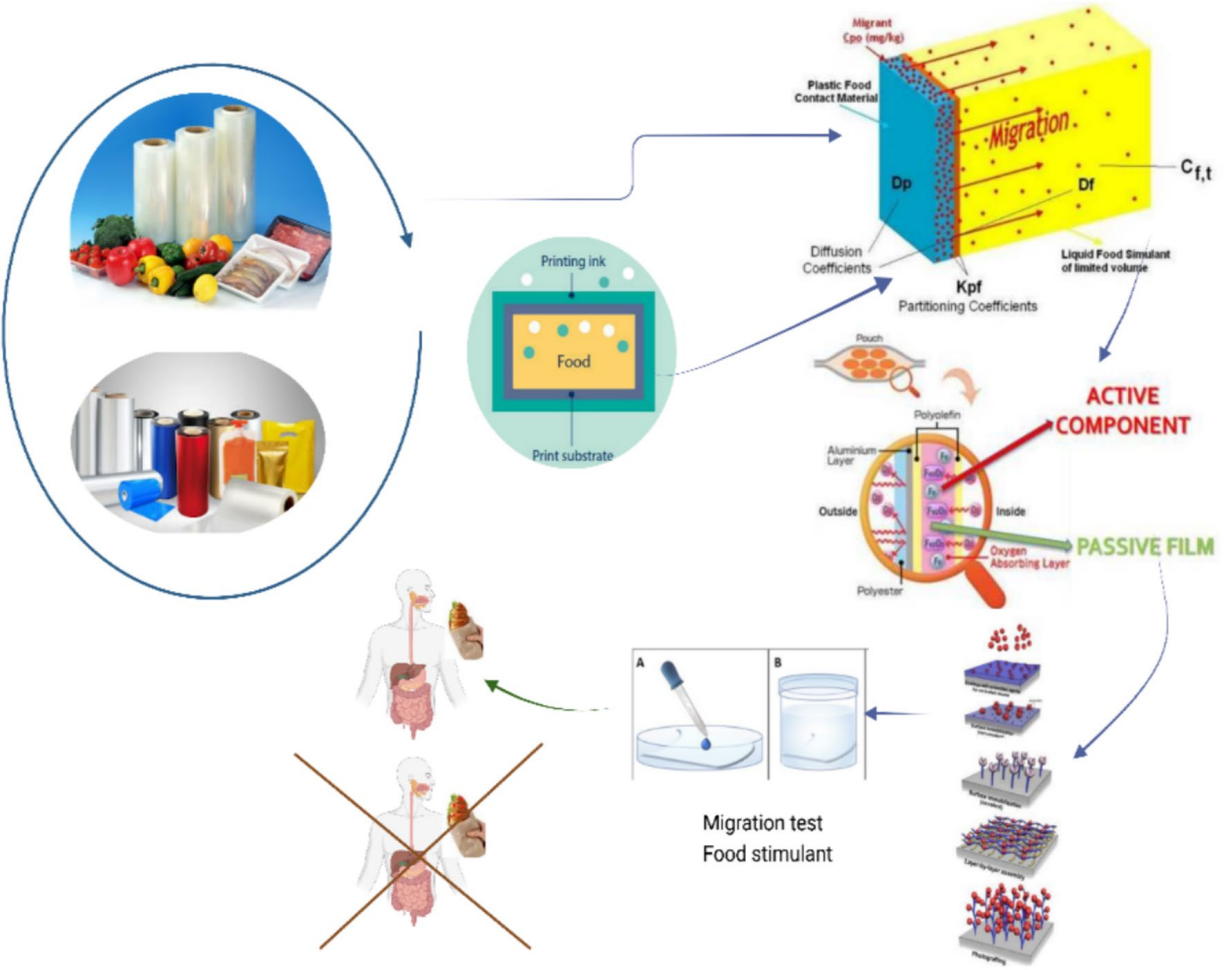
Graphic abstract

Hazard identification and assessment are essential principles in all HACCP systems. The food safety team implemented a structured methodology for hazard analysis, examining every step from receiving raw materials to product delivery. Hazards were classified into biological (illness-causing), chemical (toxic or reactive), and physical (foreign matter) risks.

Rather than relying solely on a simplified 1-to-3 scale, the assessment integrated a semi-quantitative scoring matrix with weighted factors, considering exposure frequency, severity of health impact, detectability, and historical data. This provided a more nuanced prioritization of hazards.

Control measures were selected accordingly. Hazards with low composite scores (HR < 2) were managed using PRPs, while stages such as raw material storage (CP1), extrusion (CP2), and lamination (CP3) were identified as control points due to their potential for contamination or material degradation.

The decision not to define CCPs based on the ISO 22000:2018 decision tree was made intentionally, as no high-severity hazards (HR ≥ 3) were observed. However, the Codex-recommended logic tree was consulted for verification, and supplementary analysis is available in the Supplementary Tables.

To validate the food safety strategy, a verification plan was established, including frequency, responsibility, and acceptable limits for each control measure.

This system allowed for real-time corrections and trend analysis, ensuring not just compliance but continuous improvement. The structured documentation includes not only procedures, but also historical performance metrics and audit trail data, enhancing traceability and accountability.

### TACCP Evaluation

TACCP programs typically include the full food production and supply chain process. Threats such as economically motivated adulteration, sabotage, and system interference were assessed through structured interviews, scenario analysis, and vulnerability scoring. The process begins with threat assessment, which involves identifying and assessing potential hazards to food safety and security. After establishing the scope of the study and creating a process flow, the TACCP team identified the threats that exist in the technological process of manufacturing food packaging (as shown in Supplementary Materials: Table 4).

From the analysis of the hazards and the assessment of the associated risks for the protection of the products, the areas that are of high and critical risk level are selected and the Food Defense Plan is developed.

When they are identified, the critical areas for security will be graphically represented on the General Location Plan specific to each work point. These security critical areas must be adequately protected to prevent unauthorized access.

Thus, the following critical areas have been identified and have stable practices and access policies of employees, visitors and contractors.

The first critical area identified is the area of raw materials and ingredients, where practices and access policies will be implemented, namely the access of foreign people that will be prohibited; only the manager/warehouse manager being allowed access.

The second area identified as critical is the production area, where the production manager receives and approves requests for access to the production area. In this regard, the person requesting the visit to the production area will fill in the declaration regarding the state of health at the access control point, access being prohibited to visitors who are recorded or who suffer from diseases found in the "List of communicable diseases". Also, visitors' access to the production area is only allowed with an accompanying person, after the hygiene rules and food safety measures have been presented.

Regarding internal violations, each employee will carry out his activity at the job for which he is hired; after any intervention by maintenance teams or maintenance service providers, the machines that have been intervened on will be checked and sanitized. At the same time, it is forbidden for workers from other workstations to intervene to change the operating parameters of the work equipment.

The last critical area is represented by the distribution area of the finished product, where a series of measures are taken such as the prohibition of access to unauthorized persons, the prohibition of works with open fire and/or smoking; access with oils, unidentified/prohibited substances or chemicals being prohibited, and access with similar products brought from outside is prohibited as well.

Employee training records, access logs, and incident reports were reviewed to ensure security policies are enforced. Internal vulnerabilities were minimized through clearly defined roles, restricted system access, and post-maintenance validations.

### Migration analysis results

The evaluation of the overall migration of the components was carried out using food simulants (Tables 1, 2), in accordance with Regulation (EU) No 10/2011 (10/2011, 2011). The investigation incorporated the subsequent simulants: simulant A—10% ethyl alcohol, simulant B—3% acetic acid, simulant D2—olive oil, and simulant E—tenax. Following the completion of the extraction phase, the obtained extract was evaporated in a water bath utilizing platinum crucibles. The final measurement of the total migration of the components was expressed in milligrams per square decimeter (mg/dm2). The concentration of GM (migration of substances from plastics) is limited by Regulation (EU) No 10/2011 (10/2011, 2011) to a maximum of 10 mg/dm2 on a contact area basis or 60 mg/kg in the simulant or food. A simple gravimetric technique is employed to measure the overall migration of any chemicals that are prone to transfer when exposed to temperature or other types of physical stress.

**Table 1:** Migration of food simulants from foils for automated packaging (2021–2023)

	Type of foil	Simulant	Year			Overall
			2021	2022	2023	
foils for automated packaging	PE	Simulant A	1.23 ± 0.09a	1.05 ± 0.06b	0.57 ± 0.12c	0.95 ± 0.30
		Simulant B	2.14 ± 0.19a	1.87 ± 0.23b	0.90 ± 0.24c	1.64 ± 0.58
		Simulant D2	2.71 ± 0.19a	2.29 ± 0.21b	1.15 ± 0.15c	2.05 ± 0.70
		Simulant E	1.10 ± 0.09a	0.92 ± 0.13b	0.45 ± 0.23c	0.82 ± 0.32
	PE/EVOH/PE	Simulant A	0.97 ± 0.07a	0.80 ± 0.14b	0.43 ± 0.11c	0.73 ± 0.25
		Simulant B	1.47 ± 0.13a	1.14 ± 0.13b	0.55 ± 0.13c	1.05 ± 0.41
		Simulant D2	2.06 ± 0.11a	1.65 ± 0.20b	0.93 ± 0.06c	1.54 ± 0.50
		Simulant E	1.43 ± 0.28a	0.91 ± 0.12b	0.36 ± 0.14c	0.90 ± 0.48
	PE/PA/EVOH/PA/PE	Simulant A	1.16 ± 0.12a	0.92 ± 0.09b	0.58 ± 0.19c	0.88 ± 0.28
		Simulant B	1.46 ± 0.16a	1.02 ± 0.14b	0.50 ± 0.05c	0.99 ± 0.42
		Simulant D2	2.13 ± 0.09a	1.62 ± 0.22b	0.84 ± 0.17c	1.53 ± 0.57
		Simulant E	1.15 ± 0.13a	0.90 ± 0.10b	0.34 ± 0.10c	0.79 ± 0.37
	PE/PA/EVOH/PE/PP	Simulant A	1.61 ± 0.16a	1.21 ± 0.17b	0.75 ± 0.20c	1.19 ± 0.40
		Simulant B	2.23 ± 0.12a	1.94 ± 0.58b	0.83 ± 0.24c	1.67 ± 0.71
		Simulant D2	3.64 ± 0.37a	2.92 ± 0.21b	2.17 ± 0.20c	2.91 ± 0.67
		Simulant E	1.21 ± 0.19b	0.84 ± 0.19b	4.43 ± 1.08a	2.16 ± 5.71
laminated foils for automatic packaging	PE/PE	Simulant A	1.17 ± 0.13a	0.92 ± 0.06b	0.48 ± 0.08c	0.86 ± 0.31
		Simulant B	1.57 ± 0.16a	1.17 ± 0.17b	0.70 ± 0.24c	1.14 ± 0.41
		Simulant D2	4.16 ± 0.12a	3.49 ± 0.27b	2.54 ± 0.46c	3.40 ± 0.75
		Simulant E	4.25 ± 0.25a	3.26 ± 0.43b	0.82 ± 0.28c	2.77 ± 1.51
	PE/BOPP	Simulant A	1.19 ± 0.11a	1.02 ± 0.18a	0.56 ± 0.16b	0.92 ± 0.31
		Simulant B	1.23 ± 0.08a	0.96 ± 0.11b	0.63 ± 0.13c	0.94 ± 0.27
		Simulant D2	3.62 ± 0.34a	2.96 ± 0.44b	2.63 ± 0.40b	3.07 ± 0.57
		Simulant E	1.88 ± 0.17a	0.93 ± 0.18b	0.40 ± 0.09c	1.07 ± 0.65
	PE/PET	Simulant A	1.19 ± 0.18a	1.24 ± 0.19a	0.65 ± 0.23b	1.03 ± 0.33
		Simulant B	1.70 ± 0.12a	1.09 ± 0.14b	0.64 ± 0.17c	1.14 ± 0.47
		Simulant D2	7.66 ± 0.39a	6.61 ± 0.31b	5.60 ± 0.37c	6.62 ± 0.93
		Simulant E	1.32 ± 0.21a	0.88 ± 0.10b	0.37 ± 0.08c	0.86 ± 0.42
	BOPP/BOPP	Simulant A	1.22 ± 0.08a	1.02 ± 0.10b	0.58 ± 0.13c	0.94 ± 0.29
		Simulant B	1.18 ± 0.10a	1.01 ± 0.07b	0.60 ± 0.09c	0.93 ± 0.26
		Simulant D2	6.98 ± 0.23a	6.23 ± 0.45b	5.31 ± 0.37c	6.17 ± 0.78
		Simulant E	1.33 ± 0.27a	0.90 ± 0.27b	0.33 ± 0.10c	0.85 ± 0.47
	CPP/BOPP	Simulant A	1.25 ± 0.17a	0.91 ± 0.11b	0.46 ± 0.09c	0.87 ± 0.35
		Simulant B	1.14 ± 0.13a	0.84 ± 0.17b	0.45 ± 0.09c	0.81 ± 0.32
		Simulant D2	8.05 ± 0.26a	6.90 ± 0.39b	5.37 ± 0.44c	6.77 ± 1.18
		Simulant E	1.27 ± 0.23a	0.95 ± 0.19b	0.35 ± 0.07c	0.86 ± 0.43
	CPP/PET	Simulant A	1.30 ± 0.05a	1.00 ± 0.11b	0.49 ± 0.12c	0.93 ± 0.36
		Simulant B	1.58 ± 0.20a	1.03 ± 0.10b	0.57 ± 0.14c	1.06 ± 0.45
		Simulant D2	6.89 ± 0.17a	5.90 ± 0.16b	4.63 ± 0.40c	5.80 ± 0.99
		Simulant E	1.57 ± 0.08a	1.04 ± 0.10b	0.39 ± 0.09c	1.00 ± 0.50
	BOPP metallized/BOPP	Simulant A	1.04 ± 0.04a	0.85 ± 0.15b	0.57 ± 0.15c	0.82 ± 0.23
		Simulant B	1.17 ± 0.08a	1.03 ± 0.09b	0.64 ± 0.20c	0.94 ± 0.26
		Simulant D2	5.47 ± 0.71a	4.23 ± 0.20b	3.69 ± 0.50c	4.46 ± 0.90
		Simulant E	1.55 ± 0.22a	0.90 ± 0.11b	0.36 ± 0.12c	0.93 ± 0.52
	BOPP metallized/PET	Simulant A	1.05 ± 0.09a	0.90 ± 0.12a	0.57 ± 0.12b	0.84 ± 0.23
		Simulant B	1.33 ± 0.08a	0.99 ± 0.06b	0.53 ± 0.08c	0.95 ± 0.35
		Simulant D2	3.63 ± 0.25a	2.88 ± 0.20b	1.58 ± 0.35c	2.70 ± 0.91
		Simulant E	1.01 ± 0.08a	0.80 ± 0.14b	0.37 ± 0.10c	0.73 ± 0.29
	CPP metallized/BOPP	Simulant A	1.39 ± 0.16a	1.01 ± 0.12b	0.49 ± 0.07c	0.96 ± 0.40
		Simulant B	2.17 ± 0.11a	1.53 ± 0.33b	0.61 ± 0.14c	1.44 ± 0.69
		Simulant D2	6.01 ± 0.21a	4.77 ± 0.32b	3.32 ± 0.23c	4.70 ± 1.16
		Simulant E	1.84 ± 0.26a	0.91 ± 0.10b	0.36 ± 0.09c	1.04 ± 0.65
	CPP metallized/PET	Simulant A	2.01 ± 0.23a	1.68 ± 0.19b	0.79 ± 0.24c	1.49 ± 0.57
		Simulant B	5.00 ± 0.20a	4.17 ± 0.20b	2.92 ± 0.43c	4.03 ± 0.92
		Simulant D2	5.12 ± 0.56a	3.89 ± 0.25b	2.08 ± 0.71c	3.69 ± 1.38
		Simulant E	2.24 ± 0.23a	0.94 ± 0.07b	0.41 ± 0.06c	1.20 ± 0.80
Triplex foils	PE/AL/PET	Simulant A	2.03 ± 0.07a	1.26 ± 0.23b	0.57 ± 0.13c	1.28 ± 0.63
		Simulant B	1.66 ± 0.11a	1.03 ± 0.16b	0.53 ± 0.10c	1.08 ± 0.49
		Simulant D2	4.30 ± 0.17a	3.43 ± 0.35b	2.56 ± 0.23c	3.43 ± 0.77
		Simulant E	1.29 ± 0.21a	0.93 ± 0.15b	0.37 ± 0.12c	0.86 ± 0.42
	PE/AL/BOPP	Simulant A	1.68 ± 0.16a	1.08 ± 0.31b	0.46 ± 0.11c	1.08 ± 0.55
		Simulant B	2.38 ± 0.20a	1.75 ± 0.38b	0.61 ± 0.14c	1.58 ± 0.79
		Simulant D2	4.30 ± 0.27a	3.20 ± 1.41b	2.03 ± 0.37c	3.18 ± 1.25
		Simulant E	1.57 ± 0.08a	0.86 ± 0.10b	0.35 ± 0.09c	0.93 ± 0.52
	PE/PET metallized/BOPP	Simulant A	1.18 ± 0.11a	0.85 ± 0.17b	0.47 ± 0.10c	0.83 ± 0.32
		Simulant B	1.28 ± 0.19a	0.87 ± 0.15b	0.48 ± 0.11c	0.88 ± 0.37
		Simulant D2	2.09 ± 0.30a	1.27 ± 0.18b	0.91 ± 0.10c	1.42 ± 0.54
		Simulant E	1.55 ± 0.35a	0.85 ± 0.13b	0.33 ± 0.10c	0.91 ± 0.56
	PE/PET metallized/PET	Simulant A	1.71 ± 0.34a	1.02 ± 0.47b	0.50 ± 0.07c	1.08 ± 0.60
		Simulant B	2.82 ± 0.31a	2.07 ± 0.08b	1.48 ± 0.18c	2.12 ± 0.60
		Simulant D2	3.80 ± 0.42a	3.00 ± 0.07b	2.10 ± 0.22c	2.96 ± 0.76
		Simulant E	1.47 ± 0.29a	0.93 ± 0.07b	0.33 ± 0.11c	0.91 ± 0.51
	PE/PAP/AL	Simulant A	2.46 ± 0.26a	1.97 ± 0.09b	0.57 ± 0.15c	1.66 ± 0.84
		Simulant B	2.29 ± 0.25a	1.95 ± 0.15a	0.80 ± 0.28b	1.68 ± 0.69
		Simulant D2	4.08 ± 0.35a	3.04 ± 1.22b	1.57 ± 0.36c	2.90 ± 1.28
		Simulant E	1.50 ± 0.20a	0.93 ± 0.13b	0.33 ± 0.13c	0.92 ± 0.51
	CPP/AL/PET	Simulant A	1.29 ± 0.18a	0.99 ± 0.12b	0.44 ± 0.09c	0.91 ± 0.38
		Simulant B	1.38 ± 0.32a	0.97 ± 0.15b	0.43 ± 0.10c	0.93 ± 0.45
		Simulant D2	2.26 ± 0.22a	1.90 ± 0.09b	0.78 ± 0.20c	1.64 ± 0.67
		Simulant E	1.12 ± 0.09a	0.86 ± 0.11b	0.25 ± 0.13c	0.74 ± 0.39
	CPP/AL/BOPP	Simulant A	2.13 ± 0.30a	1.73 ± 0.37b	0.52 ± 0.09c	1.46 ± 0.75
		Simulant B	2.22 ± 0.33a	1.58 ± 0.18b	0.60 ± 0.11c	1.46 ± 0.72
		Simulant D2	3.39 ± 0.52a	2.74 ± 0.30b	1.19 ± 0.47c	2.44 ± 1.04
		Simulant E	1.14 ± 0.15a	0.82 ± 0.09b	0.23 ± 0.13c	0.73 ± 0.40

The measurements are presented as the mean value plus or minus the standard deviation of three repeated measurements. The values (mean ± SD) from a row that have the same letter are not statistically significant (p > 0.05). Legend: Simulant A contains 10% ethyl alcohol, Simulant B contains 3% acetic acid, Simulant D2 is olive oil, Simulant E is tenax. The packaging materials include polyethylene foil (PE), ethylene copolymer and vinyl alcohol foil (EVOH), polyamide foil (PA), polypropylene foil (PP), biaxially oriented polypropylene foil (BOPP), cast polypropylene film (CPP), metalized biaxially oriented polypropylene (BOPP metalized), and metalized cast polypropylene (CPP metalized), polyethylene terephthalate foil (PET), aluminum foil (AL), metalized polyethylene terephthalate (PET metalized), and paper foil (PAP)

**Table 2:** Microbiological analysis for foils (2021–2023)

	Type of foil	Characteristics	U.M	Year			Reference values	
				2021	2022	2023		
Foils for automated packaging	PE	Coliform bacteria	CFU/cm2	Absent	Absent	Absent	Absent	
		Total bacterial count	CFU/cm^2^	< 1	< 1	< 1	1	
		Molds	CFU/cm^2^	< 1	< 1	< 1	3	
	PE/EVOH/PE	Coliform bacteria	CFU/cm2	Absent	Absent	Absent	Absent	
		Total bacterial count	CFU/cm^2^	< 1	< 1	< 1	1	
		Molds	CFU/cm^2^	< 1	< 1	< 1	3	
	PE/PA/EVOH/PA/PE	Coliform bacteria	CFU/cm2	Absent	Absent	Absent	Absent	
		Total bacterial count	CFU/cm^2^	< 1	< 1	< 1	1	
		Molds	CFU/cm^2^	< 1	< 1	< 1	3	
	PE/PA/EVOH/PE/PP	Coliform bacteria	CFU/cm2	Absent	Absent	Absent	Absent	
		Total bacterial count	CFU/cm^2^	< 1	< 1	< 1	1	
		Mold detection	CFU/cm^2^	< 1	< 1	< 1	3	
Laminated foils for automatic packaging	PE/PE	Coliform bacteria	CFU/cm^2^	Absent	Absent	Absent	Absent	
		Total bacterial count	CFU/cm^2^	< 1	< 1	< 1	1	
		Molds	CFU/cm^2^	< 1	< 1	< 1	3	
	PE/BOPP	Coliform bacteria	CFU/cm^2^	Absent	Absent	Absent	Absent	
		Total bacterial count	CFU/cm^2^	< 1	< 1	< 1	1	
		Molds	CFU/cm^2^	< 1	< 1	< 1	3	
	PE/PET	Coliform bacteria	CFU/cm^2^	Absent	Absent	Absent	Absent	
		Total bacterial count	CFU/cm^2^	< 1	< 1	< 1	1	
		Molds	CFU/cm^2^	< 1	< 1	< 1	3	
	BOPP/BOPP	Coliform bacteria	CFU/cm^2^	Absent	Absent	Absent	Absent	
		Total bacterial count	CFU/cm^2^	< 1	< 1	< 1	1	
		Molds	CFU/cm^2^	< 1	< 1	< 1	3	
	CPP/BOPP	Coliform bacteria	CFU/cm^2^	Absent	Absent	Absent	Absent	
		Total bacterial count	CFU/cm^2^	< 1	< 1	< 1	1	
		Molds	CFU/cm^2^	< 1	< 1	< 1	3	
	CPP/PET	Coliform bacteria	CFU/cm^2^	Absent	Absent	Absent	Absent	
		Total bacterial count	CFU/cm^2^	< 1	< 1	< 1	1	
		Molds	CFU/cm^2^	< 1	< 1	< 1	3	
	BOPP metallized/BOPP	Coliform bacteria	CFU/cm^2^	Absent	Absent	Absent	Absent	
		Total bacterial count	CFU/cm^2^	< 1	< 1	< 1	1	
		Molds	CFU/cm^2^	< 1	< 1	< 1	3	
	BOPP metallized/PET	Coliform bacteria	CFU/cm^2^	Absent	Absent	Absent	Absent	
		Total bacterial count	CFU/cm^2^	< 1	< 1	< 1	1	
		Molds	CFU/cm^2^	< 1	< 1	< 1	3	
	CPP metallized/BOPP	Coliform bacteria	CFU/cm^2^	Absent	Absent	Absent	Absent	
		Total bacterial count	CFU/cm^2^	< 1	< 1	< 1	1	
		Molds	CFU/cm^2^	< 1	< 1	< 1	3	
	CPP metallized/PET	Coliform bacteria	CFU/cm^2^	Absent	Absent	Absent	Absent	
		Total bacterial count	CFU/cm^2^	< 1	< 1	< 1	1	
		Molds	CFU/cm^2^	< 1	< 1	< 1	3	
Triplex foils	PE/AL/PET	Coliform bacteria	CFU/cm^2^	Absent	Absent	Absent	Absent	
		Total bacterial count	CFU/cm^2^	< 1	< 1	< 1	1	
		Molds	CFU/cm^2^	< 1	< 1	< 1	3	
	PE/AL/BOPP	Coliform bacteria	CFU/cm^2^	Absent	Absent	Absent	Absent	
		Total bacterial count	CFU/cm^2^	< 1	< 1	< 1	1	
		Molds	CFU/cm^2^	< 1	< 1	< 1	3	
	PE/PET metallized/BOPP	Coliform bacteria	CFU/cm^2^	Absent	Absent	Absent	Absent	
		Total bacterial count	CFU/cm^2^	< 1	< 1	< 1	1	
		Molds	CFU/cm^2^	< 1	< 1	< 1	3	
	PE/PET metallized/PET	Coliform bacteria	CFU/cm^2^	Absent	Absent	Absent	Absent	
		Total bacterial count	CFU/cm^2^	< 1	< 1	< 1	1	
		Molds	CFU/cm^2^	< 1	< 1	< 1	3	
	PE/PAP/AL	Coliform bacteria	CFU/cm^2^	Absent	Absent	Absent	Absent	
		Total bacterial count	CFU/cm^2^	< 1	< 1	< 1	1	
		Molds	CFU/cm^2^	< 1	< 1	< 1	3	
	CPP/AL/PET	Coliform bacteria	CFU/cm^2^	Absent	Absent	Absent	Absent	
		Total bacterial count	CFU/cm^2^	< 1	< 1	< 1	1	
		Molds	CFU/cm^2^	< 1	< 1	< 1	3	
	CPP/AL/BOPP	Coliform bacteria	CFU/cm^2^	Absent	Absent	Absent	Absent	
		Total bacterial count	CFU/cm^2^	< 1	< 1	< 1	1	
		Molds	CFU/cm^2^	Absent	Absent	Absent	3	

According to the IFS Packaging Guideline, version 2 released in 2023, migration is the process of transferring chemicals and/or components from the packaging to the food. The movement of these elements is limited in most nations due to their potential to cause harm to human health. The migration of compounds from food contact materials is affected by multiple factors, such as the food's composition (including fats and acids), the nature of the interaction (whether direct or indirect), the barrier properties of the materials, the duration of contact, the temperature during contact, the characteristics of the packaging material, the properties of the migrating substance, and the quantity of the migrating substance present in the packaging material (IFS PACsecure Standard (version 2, 2023).

After analyzing the data on the migration of each simulant tested for various foils and films described earlier, disparities were identified between the investigated types of foils and films in relation to their application in the food industry.

The data analysis revealed considerable disparities in both the study years and the tested materials for films used in automated packing. Across all categories of foils and simulants, there was a decline in the reported values from 2021 to 2023, suggesting a decrease in the migration of the compound with time (Table 1). It was noted that various types of foils exhibited varying degrees of compound migration. For example, the migration levels differed between PE foil and PE/EVOH/PE foils. The utilization of barrier materials (specifically EVOH) has not only enhanced the overall quality of foils in recent years, but it also has the potential to significantly diminish or totally prevent the transfer of substances from the outer layer to the food by the process of diffusion (Schmid and Welle, 2020). The outcomes of our investigation confirm that this plays a pivotal role in enhancing the safety of the product. The analysis of the data also uncovered the impact of different simulants on the movement of the chemical. More precisely, the migration rate in simulant D2 (olive oil) was demonstrated to be higher than that in simulant A (10% ethyl alcohol). The observed decline in simulant migration from the tested material across a span of three years indicates a consistent enhancement in the quality of the packaging materials. This is supported by a decrease in the rate of compound migration, along with an increase in production resulting from more stringent quality control methods and adherence to food safety and packaging requirements.

Laminated foils for automatic packaging, similarly to foils for automatic packaging, showed an overall downward tendency from 2021 to 2023 (Table 1). Certain simulants, such as D2 (olive oil) and E (tenax), exhibited a particularly noticeable downward migration trend in the types of films analyzed, suggesting that these materials either had a higher initial migration rate or that packaging technology and processes have improved. In the context of a quality management system on the safety of laminated and unlaminated films in the food industry, the decrease in migration values over time indicates an improved manufacturing process, which includes fewer contaminants and more stable materials and compliance with the European food safety packaging regulations.

The data for the triplex foils, which belong to the final category analyzed, are expressed in the same manner as the other two categories presented below. The parameters analyzed during the period from 2021 to 2023, for migration of the food simulants mentioned in the table, are presented as mean values with their corresponding standard deviations (Table 1). This representation indicates the level of variability for each measurement. Therefore, the analysis demonstrates a consistent decline in the values of migration of food simulants in all packaging materials throughout 2021–2023. The values reported in 2021 for all examined packaging materials for the tested simulants are the highest. They fall in 2022 and retain the same pattern in 2023. An overall decrease in the values of all three categories of tested materials is noted throughout the study. The standard deviation has shifted along with this decrease, indicating a variation in the findings reported in all three tables. The acquired results were within the permissible limit for each simulant, indicating that the packing materials comply with the global migration of the components. Similar findings were also reported by Ungureanu (Ungureanu, 2021).

A significant finding is that even in cases where migration values decreased, variability among batches and materials was observed, highlighting the importance of ongoing monitoring, not just compliance verification.

### Microbiologic testing

According to Regulation (EC) no. 178/2002 (178/2002, 2002), operators in the food sector are tasked with the obligation to ensure the safety and adherence to regulations of both the food products and the packaging materials that come into touch with them. They must provide confirmation of the safety of the packaging materials and ensure that all participants in the supply chain can prove the safety of items meant for human consumption. Therefore, concerning the microbiological management of the packing material units, quarterly tests were conducted for three years. These tests specifically focused on coliform bacteria, the total bacterial count, and mold detection (Table 2). The surveillance of these three primary criteria was conducted following the requirements outlined in the Order of the Minister of Health no. 976/1998 (976/1998, 1998), which pertains to the certification of food hygiene standards in Romania. This surveillance encompasses the entire food supply chain, including production, processing, transportation, storage, and marketing. Their measurement was performed in colony-forming units per square centimeter (CFU/cm^2^). The analysis of films from 2021–2023 revealed that there is no presence of coliform bacteria. The total number of germs and molds was very low and complied with the current legislation (less than 1 CFU/cm^2^). Thus, during the studied period this can be considered an indicator that there is an effective control (HACCP plan) in place to prevent microbiological contamination of these types of food contact materials. These results can be obtained and improved through a food safety and security system, a controlled atmosphere and adequate temperature (Delgado et al. 2023; Guynot et al., 2003; Omerović et al., 2021).

Food foils are extensively utilized in the food business to provide chemical, biological, and physical safeguards for food products, preventing spoiling and damage at every stage of the food supply chain. Therefore, its purpose is to uphold the standard of the product, establishing a crucial setting for safeguarding the quality of the packed item. The current investigation tracked the movement of substances from laminated and unlaminated food films, employing standardized food stimulants in controlled test circumstances, alongside a microbiological examination. The results achieved remained within the permissible limit for each simulant, exhibiting a decline over the course of the three-year investigation. This indicates that the packing materials adhere to the overall migration standards of the components and the implemented food safety protocols. Hence, the findings obtained throughout the study period underscore the importance of incorporating hygiene and quality control in the production process and ensuring adherence to standards for materials that come into contact with food items. Integrated systems are crucial in minimizing the probability of microbiological contamination, ensuring the safety of the food chain, and preserving the quality of both laminated and non-laminated films.

## Supplementary Information

Below is the link to the electronic supplementary material.

Supplementary file1 (DOCX 279 KB)
