# Supplementary material for: An assessment of the influence of elevated hygiene hazards and quality management systems on the safety of laminated and unlaminated films employed in the food sector
Source: Food Sci Biotechnol. 2025 Jul 24;34(14):3403–15. doi: 10.1007/s10068-025-01926-8 (PMC12408432; doi:10.1007/s10068-025-01926-8)
Supplement: Supplementary file 1 — Supplementary file1 (DOCX 279 KB) [file 10068_2025_1926_MOESM1_ESM.docx]

**Supplementary Materials**


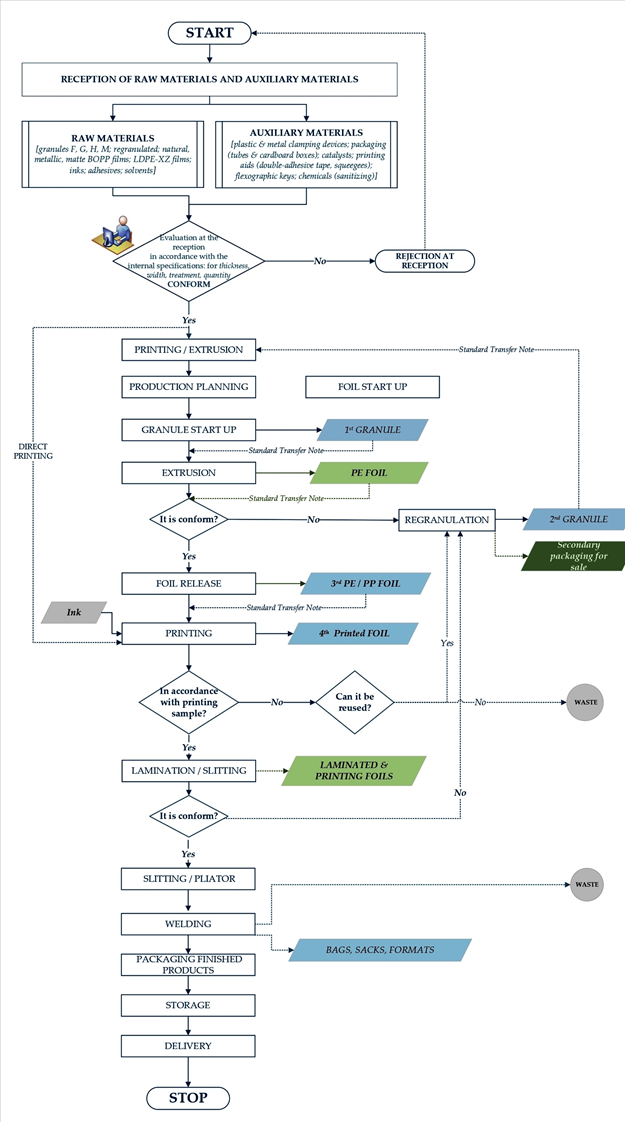


**Figure 1**. Flow chart

**Table 1.** Hazard analysis and assessment

| **The step of the**  **technological process** | **Identify potential hazards introduced, controlled, or improved at this step** | | **Does this potential hazard need to be addressed in the HACCP plan?**  **Yes/No** | **Justify your decision** | **Hazard assessment** | | | **What measure(s) can be applied to prevent or eliminate the hazard or reduce in to an acceptable level?** |
| --- | --- | --- | --- | --- | --- | --- | --- | --- |
|  |  |  |  |  | **S** | **P** | **HR** |  |
| General, for all steps | **B** | Bacteria,  Viruses  Protozoa  Pathogenic agents producing thermostable toxins (Bacteria, Molds) | Yes | Contamination can lead to obtaining an inappropriate product | 2 | 1 | 2 | - applying disinfection and hand hygiene rules for all personnel upon entering the unit, wearing a face mask and disposable gloves; - compliance with the principle "FEFO" and stock rotation; - appropriate hand hygiene, according to the Hand Washing SOP (changing min. 1x/hour of the dirty gloves for the people handling materials or when necessary, plus washing and disinfecting hands at entering each time the production area); - wearing appropriate work equipment for protection (white for production employees and dark blue for the maintenance staff), with a minimum of three complete rows of equipment for each employee; - washing of all work equipment by an external service provider (1x/week collecting); - checking the washing efficiency for the work equipment by taking internal sanitation tests for the washed protective equipment 1 x/week (RLU reading → directly proportional to the amount of ATP collected from the sample) and externally, accreditating ISO 17025 test reports at a frequency of 2 x/year for the following parameters: aerobic total viable count (TVC) and coliforms; - restricting staff access to the areas in accordance with the job description; - personal training with the specific SOP (Standard Operational Procedures), as follows: HACCP system and CCP monitoring, personal hygiene, sanitation program, measuring and monitoring devices, production, reception, storage, allergen management, food fraud, food defense, foreign body management, and management of transport; - writing the technical specifications and establishing acceptability criteria for raw materials; - analysis of the product in accordance with established criteria (legal base); - evaluation of suppliers according to internal supply / purchase procedures; - training of purchase department employees in order to understand and respect the acceptability criteria; - application of the provisions of the allergen management procedure and vulnerability study; - identification of allergenic products according to Reg. E.U. 1169/2011 (at reception, if that is the case); - re-evaluation of suppliers where non-conformities were identified at the reception; - removing from the list of accepted suppliers those who do not meet the acceptance conditions established by the supply procedure after complaint management and recurrence of the same issue; |
|  | **C** | Compounds of natural origin  Chemical substances in ingredients  Environmental pollutants  Process additives  Process chemicals  Packaging materials  Acts of sabotage | Yes | The presence of chemical products on the product can lead to obtaining an inappropriate product | 1 | 1 | 1 |  |
|  | **P** | Products of vegetable / organic origin. Foreign bodies made of metal, plastic, glass, wood, etc.  Infestation with insects, rodents  Signs of rodent attack  Plastic  Impurities from machine maintenance  Personal belongings of employees | Yes | It can lead to obtaining an inappropriate product | 1 | 1 | 1 |  |
|  | **A** | Allergens | Yes | It can lead to comsumer health impact | 1 | 1 | 1 |  |
| Qualitative and quantitative reception of raw materials and auxiliary materials | **B** | Microbiological contamination | Yes | Contamination can lead to obtaining an inappropriate product | 1 | 1 | 1 | - purchase of goods only from evaluated suppliers - the receipt of the goods is done only if it is accompanied by a declaration of conformity   qualitative and quantitative inspection of the cargo lot   - checking the state of hygiene of means of transport |
|  | **C** | Contamination with chemical substances | Yes | The presence of chemical products on the product can lead to obtaining an inappropriate product | 1 | 1 | 1 |  |
|  | **P** | The presence of foreign, harmful bodies | Yes | It can lead to obtaining an inappropriate product | 1 | 1 | 1 |  |
| Storage of raw materials and auxiliary materials | **B** | Proliferation of microorganisms | Yes | Proliferation can lead to obtaining an inappropriate product | 2 | 1 | 2 | - compliance with GMP measures - personal training regarding personal hygiene and during work - maintaining the temperature within the limits of 5-30°C and the humidity within the limits of 40-60% - temperature and humidity monitoring in manual storage spaces |
|  | **C** | Contamination with washing chemicals (failure to observe the detergent concentration, improper rinsing) | Yes | The presence of chemical products on the product can lead to obtaining an inappropriate product | 1 | 1 | 1 |  |
|  | **P** | The presence of foreign, harmful bodies | Yes | Can lead to obtaining an inappropriate product | 1 | 1 | 1 |  |
| Unpacking and preparation of raw materials and materials | **B** | Microbiological contamination | Yes | Contamination can lead to obtaining an inappropriate product | 1 | 1 | 1 | - compliance with GMP measures - personal training regarding personal hygiene and during work |
|  | **C** | Contamination with chemical substances | Yes | the presence of chemical products on the product can lead to obtaining an inappropriate product | 1 | 1 | 1 |  |
|  | **P** | The presence of foreign, harmful bodies | Yes | Can lead to obtaining an inappropriate product | 1 | 1 | 1 |  |
| Extrusion | **B** | Microbiological contamination | Yes | Contamination can lead to obtaining an inappropriate product | 2 | 1 | 2 | - compliance with GMP measures - personal training regarding personal hygiene and during work - extrusion temperature monitoring depending on the type of product - sanitation and aeromicroflora tests according to the self-control plan - periodic machine check - periodic machine sanitization |
|  | **C** | Contamination with chemical substances | Yes | The presence of chemical products on the product can lead to obtaining an inappropriate product | 1 | 1 | 1 |  |
|  | **P** | The presence of foreign, harmful bodies | Yes | Can lead to obtaining an inappropriate product | 1 | 1 | 1 |  |
| Printing | **B** | Microbiological contamination | Yes | Contamination can lead to obtaining an inappropriate product | 1 | 1 | 1 | - compliance with GMP measures - personal training regarding personal hygiene and during work - sanitation and aeromicroflora tests according to the self-control plan - compliance with the typology/cromalines - periodic machine check - periodic machine sanitization - compliance check of printed products |
|  | **C** | Contamination with chemical substances | Yes | The presence of chemical products on the product can lead to obtaining an inappropriate product | 1 | 1 | 1 |  |
|  | **P** | The presence of foreign, harmful bodies | Yes | Can lead to obtaining an inappropriate product | 1 | 1 | 1 |  |
| Lamination (Rolling) | **B** | Microbiological contamination | Yes | Contamination can lead to obtaining an inappropriate product | 2 | 1 | 2 | - compliance with GMP measures - personal training regarding personal hygiene and during work - periodic machine check - periodic machine sanitization - compliance check of laminated products |
|  | **C** | Contamination with chemical substances | Yes | The presence of chemical products on the product can lead to obtaining an inappropriate product | 1 | 1 | 1 |  |
|  | **P** | The presence of foreign, harmful bodies | Yes | Can lead to obtaining an inappropriate product | 1 | 1 | 1 |  |
| Debiting | **B** | Microbiological contamination from personnel and equipment | Yes | Contamination can lead to obtaining an inappropriate product | 1 | 1 | 1 | - compliance with GMP measures - personal training regarding personal hygiene and during work - periodic machine check - periodic machine sanitization - compliance check of debited products - migration tests for raw materials according to the self-control plan |
|  | **C** | Contamination with chemical substances | Yes | The presence of chemical products on the product can lead to obtaining an inappropriate product | 1 | 1 | 1 |  |
|  | **P** | The presence of foreign, harmful bodies | Yes | Can lead to obtaining an inappropriate product | 1 | 1 | 1 |  |
| Welding | **B** | Microbiological contamination | Yes | Contamination can lead to obtaining an inappropriate product | 1 | 1 | 1 | - compliance with GMP measures - personal training regarding personal hygiene and during work - sanitation and aeromicroflora tests according to the self-control plan - migration tests for raw materials according to the self-control plan - periodic machine check - periodic machine sanitization - verification of compliance of welded products |
|  | **C** | Contamination with chemical substances | Yes | The presence of chemical products on the product can lead to obtaining an inappropriate product | 1 | 1 | 1 |  |
|  | **P** | The presence of foreign, harmful bodies | Yes | Can lead to obtaining an inappropriate product | 1 | 1 | 1 |  |
| Packaging, labeling, palletizing | **B** | Microbiological contamination | Yes | Contamination can lead to obtaining an inappropriate product | 1 | 1 | 1 | - compliance with GMP measures - personal training regarding personal hygiene and during work |
|  | **C** | Contamination with chemical substances | Yes | The presence of chemical products on the product can lead to obtaining an inappropriate product | 1 | 1 | 1 |  |
|  | **P** | The presence of foreign, harmful bodies | Yes | Can lead to obtaining an inappropriate product | 1 | 1 | 1 |  |
| Storage | **B** | Microbiological contamination | Yes | Contamination can lead to obtaining an inappropriate product | 2 | 1 | 2 | - compliance with GMP measures - personal training regarding personal hygiene and during work - maintaining the temperature within the limits of 5-30°C and the humidity within the limits of 40-60% - temperature and humidity monitoring in manual storage spaces |
|  | **C** | Contamination with chemical substances | Yes | The presence of chemical products on the product can lead to obtaining an inappropriate product | 1 | 1 | 1 |  |
|  | **P** | The presence of foreign, harmful bodies | Yes | Can lead to obtaining an inappropriate product | 1 | 1 | 1 |  |
| Delivery | **B** | Microbiological contamination | Yes | Contamination can lead to obtaining an inappropriate product | 1 | 1 | 1 | - checking the state of hygiene of means of transport |
|  | **C** | Contamination with chemical substances | Yes | The presence of chemical products on the product can lead to obtaining an inappropriate product | 1 | 1 | 1 |  |
|  | **P** | The presence of foreign, harmful bodies | Yes | Can lead to obtaining an inappropriate product | 1 | 1 | 1 |  |

**Table 2**. CCP / CP identification

| **Process step** | **Significant hazard** | **Q1^1^** | **Q2^2^** | **Q3^3^** | **Q4^4^** | **CCP/**  **CP**  **YES / NO** |
| --- | --- | --- | --- | --- | --- | --- |
| Qualitative and quantitative reception of raw materials and auxiliary materials | **B:** microbiological contamination from the supplier | Yes | No | Yes | No | **PRPO 1** |
|  | **C:** contamination with chemicals from the means of transport | Yes | No | Yes | No |  |
|  | **F:** presence of foreign, harmful bodies | Yes | No | Yes | No |  |
| Storage of raw materials and auxiliary materials | **B**: Microbiological contamination due to inappropriate temperature and humidity; due to non-compliance with GMP measures | Yes | No | No | - | **CP 1** |
|  | **C**: contamination with chemicals from washing | Yes | No | No |  |  |
|  | **F**: presence of foreign, harmful bodies | Yes | No | No |  |  |
| Unpacking and preparation of raw materials and materials | **B**: microbiological contamination due to non-compliance with GMP measures | Yes | No | Yes | No | **PRPO 2** |
|  | **C**: contamination with chemicals from washing | Yes | No | Yes | No |  |
|  | **F**: presence of foreign, harmful bodies | Yes | No | Yes | No |  |
| Extrusion | **B**: microbiological contamination due to non-compliance with GMP measures | Yes | No | No | - | **CP 2** |
|  | **C**: contamination with chemicals from washing | Yes | No | No |  |  |
|  | **F**: presence of foreign, harmful bodies | Yes | No | No |  |  |
| Printing | **B**: microbiological contamination due to non-compliance with GMP measures | Yes | No | Yes | No | **PRPO 3** |
|  | **C**: contamination with chemicals from washing | Yes | No | Yes | No |  |
|  | **F**: presence of foreign, harmful bodies | Yes | No | Yes | No |  |
| Lamination (Rolling) | **B**: microbiological contamination due to non-compliance with GMP measures | Yes | No | No | - | **CP 3** |
|  | **C**: contamination with chemicals from washing | Yes | No | No |  |  |
|  | **F**: presence of foreign, harmful bodies | Yes | No | No |  |  |
| Debiting | **B**: microbiological contamination due to non-compliance with GMP measures | Yes | No | Yes | No | **PRPO 4** |
|  | **C**: contamination with chemicals from washing | Yes | No | Yes | No |  |
|  | **F**: presence of foreign, harmful bodies | Yes | No | Yes | No |  |
| Welding | **B**: microbiological contamination due to non-compliance with GMP measures | Yes | No | Yes | No | **PRPO 5** |
|  | **C**: contamination with chemicals from washing | Yes | No | Yes | No |  |
|  | **F**: presence of foreign, harmful bodies | Yes | No | Yes | No |  |
| Packaging, labeling, palletizing | **B**: microbiological contamination due to non-compliance with GMP measures | Yes | No | Yes | No | **PRPO 6** |
|  | **C**: contamination with chemicals from washing | Yes | No | Yes | No |  |
|  | **F**: presence of foreign, harmful bodies | Yes | No | Yes | No |  |
| Storage | **B**: The proliferation of microorganisms as a result of non-compliance with temperature and humidity | Yes | No | No | - | **CP 4** |
|  | **C**: contamination with chemicals from washing | Yes | No | No |  |  |
|  | **F**: presence of foreign, harmful bodies | Yes | No | No |  |  |
| Delivery | **B**: microbiological contamination due to non-compliance with GMP measures | Yes | No | Yes | No | **PRPO 7** |
|  | **C**: contamination with chemicals from washing | Yes | No | Yes | No |  |
|  | **F**: presence of foreign, harmful bodies | Yes | No | Yes | No |  |

**Table 3.** Establishing verification procedures

| **No. crt.** | **Field of verification / item** | **Frequency** | **Responsible for verification** |
| --- | --- | --- | --- |
| 1. | Verification of compliance with the procedure for selecting suppliers; | Annual or at introduction of a new supplier in the system | Purchase Responsible |
| 2. | Checking the conformity of transport at reception (daily or each reception) and at delivery (each delivery); | Daily or each reception / each transport | Stockkeeper  Logistic responsible |
| 3. | Checking the quality of raw materials | Annual, biannual and / or monthly | HACCP team leader |
| 4. | Checking the temperature and hygiene conditions from raw material storage warehouses and final product warehouse; | Daily / as long the product is kept into the storage or transported | Logistic Responsible  Production Responsible  Stockkeeper |
| 5. | Potable water supply check | Annual | Hygiene Responsible |
| 6. | Verification of compliance with the stages of the technological flow | Monthly | Technological engineer |
| 7. | Verification of compliance with equipment maintenance | Annual, biannual and / or monthly | Maintenance manager |
| 8. | Verification of calibration of measuring and control device | Annual or when it is necessary. | Maintenance responsible |
| 9. | Checking the hygiene of production protective equipment, spaces, annexes, and social groups | Internal (weekly)  External (1x/ 3 months) | Hygiene Responsible  HACCP Team leader |
| 10. | Checking the control of the health of the staff | Biannual | Production Responsible |
| 11. | Checking the hygiene of the work equipment | Internal (weekly)  External (1x/ 3 months) | Hygiene Responsible  HACCP Team leader |
| 12. | Checking efficiency for waste disposal | Monthly | HACCP team leader |
| 13. | Verification of compliance with the pest control procedure | Monthly | Hygiene Responsible |
| 14. | Verification of CP records; deviations from critical limits; execution of corrections and / or corrective actions | Daily | HACCP team leader |
| 15. | Checking CP records | Daily | Production responsible  HACCP team leader |
| 15. | Checking the efficiency of employees training | Once every three months | HR Manager  Production Responsible  HACCP team leader |
| 16. | Checking the quality control of final products | Internal (daily)  External (monthly) | Production Responsible  HACCP team leader |
| 17. | Checking the registration activity | Monthly | HACCP team secretary |
| 18. | Checking the registration and settlement mode of complaints, trend analysis conclusions | Monthly | HACCP team leader |
| 19. | Checking team biovigilance | Annual | TACCP team |

*Evaluating TACCP plan*

The threats that have been identified can be carefully appraised for their impact and likelihood. Threats can be risked and graded based on the likelihood and severity of their occurrence. The food packaging production facility used a 3 x 3 matrix for threat assessments. Using the threat matrix, these are identified as NORMAL (low risk: from 1 to 3), HEIGHTENED (moderate risk: from 3 to 4), or CRITICAL (high risk: from 6 to 9). Each possible threat is rated on a scale of 1 to 3. A threat is considered critic if its threat rating (TR), calculated by multiplying the likelihood by the severity, above 3.

Following the application of the TACCP plan, the following decision criteria are taken into account, depending on the threat class:

- 9 - Monitoring/doubling security; Re-evaluation of the security service / termination of the security contract and subcontracting of a new security service / assessment and analysis of physical security risks;
- 6 – Over-control regarding the access to the unit of all categories of personnel and vehicles. Maximum restriction of visitors, re-evaluation of suppliers and service providers;
- 4 – Limiting access to critical areas, prohibiting access without approval or without a companion from the concerned compartment, risk reassessment;
- 3 – Guard retraining, monitoring the fulfillment of security guard duties, retraining project managers, managers regarding the supervision of external personnel (service providers, suppliers);
- 1 - 2 – No further measures are required

**Table 4:** Threats analysis and assessment

| **Process stage** | **Potential hazard identified** | **Probability** | **Severity** | **Threat risk** | **Accessibility** | **The justification of the hazard** | **Impact** | **Control measures / Corrective actions** | **Responsible** |
| --- | --- | --- | --- | --- | --- | --- | --- | --- | --- |
| External: Closing / protecting the company | An outsider can easily enter the company because the security guard fails to close the gate/barrier | 2 | 2 | 4 | easy | Intrusion by unauthorized personnel/product contamination | Medium impact  The difference depends on the type of contamination or raw material | 1. Closing the barrier/gate after every vehicle that enters/leaves the organization. | - security agent |
|  |  |  |  |  |  |  |  | 2. Regular training of security guards regarding the access of foreign persons | - guard responsible |
| External: Closing / protecting the company | An outsider can easily enter the company because the security guard cannot supervise the entire perimeter of the objective | 1 | 2 | 2 | difficult | Penetration of unauthorized personnel/ product contamination | Medium impact  The difference depends on the type of contamination or raw material | Installation of perimeter surveillance cameras and monitor in PCA  The perimeter of the company is closed with a high fence, and the external doors of the factory are permanently locked) | IT manager;  mechanics on shift |
| External: Closing / protecting the company | An external person can easily enter the company with the vehicle because the barrier is automatically raised upon recognition of the registration number of the vehicles of partners or own employees. | 2 | 2 | 4 | easy | Intrusion by unauthorized personnel/product contamination | Medium impact  The difference depends on the type of contamination or raw material | Training drivers of partners / service providers and employees to stop for access control | -department managers |
| External: Closing / protecting the company | An outsider can easily enter the building and move freely because he is not accompanied from entering the company | 2 | 2 | 4 | Easy for outside/difficult for inside | Intrusion by unauthorized personnel/product contamination | Medium impact  The difference depends on the type of contamination or raw material | Prohibition of access to the organization without a companion | - security agent |
|  |  |  |  |  |  |  |  | Supervision of service providers during the performance of the works |  |
| External: Closing / protecting the company | Breaks in the fence, doors and/or windows left open | 1 | 2 | 2 | difficult | Intrusion by unauthorized personnel | Medium impact – impossibility of packaging and delivery | At the end of every production, the guards go around the yard and check if the doors and windows are closed. If they are open, notify the responsible persons (resp. security, team leader, managers, etc.). Any break in the fence is reported to the security guard and the maintenance department. | - security agent |
| External: Access to individuals | Unauthorized and unapproved entry at the access control point | 1 | 2 | 2 | difficult | Intrusion by unauthorized personnel/product contamination | Intrusion by unauthorized personnel/product contamination | 1. All persons are authorized to enter the company regardless of whether they have a car or not.  Upon the arrival of a visitor, the guard notifies the concerned person who gives him or her an agreement to enter.  The cars that have the consent of those in charge of the process to enter the unit are registered in the guards register with the time of entry/exit from the unit. | - security agent |
|  |  |  |  |  |  |  |  | 2. Any new employee is registered by a security guard for later identification. All employees enter the company through the same place (on the main gate) and are recognized by the guards. |  |
| Internal: Personal | Employees can move without restrictions from the work area where they are assigned to other areas | 2 | 2 | 4 | easy | Intrusion by unauthorized personnel/product contamination | Medium impact – impossibility of packaging and delivery | Leaving the workplace only with the approval of the workplace manager (shift manager, packaging manager, formation manager) | Production Manager, Head of Band |
|  |  |  |  |  |  |  |  | - Prohibition of access to unauthorized persons in the kneading area, modeling division |  |
| Internal: Personal | Employees have access to the company and outside of working schedule | 1 | 3 | 3 | easy | Intentional damage / contamination | It can be big - impact on the health of the consumer, impossibility of packaging and delivery, decreased credibility on the market | Staff access outside of business hours is only with the approval of the department manager and referral to the security guard. | Department manager |
| Internal: Personal | Intentional access with:  - toxic and flammable substances  - prohibited substances (alcohol, drugs, etc.) - white weapons and sharp objects | 2 | 3 | **6** | difficult | Intentional contamination of raw materials or finished product; Chemical hazard: - spillage of chemical substances - intentional | It can be big  - impact on the health of the consumer, impossibility of packaging and delivery, decreased credibility on the market | Checking the presentation of the employees during the work shift and taking them from the changing rooms; - baggage control upon entering the company;  -testing of personnel who show signs of alcohol or drug consumption;  -retention at PCA of blunt objects (white weapons and sharp objects); - the prohibition of entering the security areas with liquids or other materials;  - video monitoring of the entire production process. | - security agent;  - production manager;  - head of formation;  - shift leader;  - the food defense team |
| External: Personal | After unwanted dismissal, staff may still have access to the company outside of business hours | 1 | 3 | 3 | difficult | Intentional damage / contamination | It can be big - impact on the health of the consumer, impossibility of packaging and delivery, decreased credibility on the market | At the termination of the individual employment contract, each employee hands over the access card and the RU representative informs the IT manager of the termination of the contract and requests the limitation of the former employee's access to the system. | - responsible for HR |
|  |  |  |  |  |  |  |  | -Legitimization of all persons upon entering the organization | - responsible for IT |
|  |  |  |  |  |  |  |  |  | -security agent |
| Service providers | Intentional access with:  - toxic and flammable substances;  - prohibited substances (alcohol, drugs, etc.);  -white weapons and sharp objects | 2 | 3 | 6 | Easy | Intentional damage / contamination;  Chemical hazard: - spillage of chemical substances - intentional | It can be big - impact on the health of the consumer, impossibility of packaging and delivery, decreased credibility on the market | - strict monitoring of service providers:  - luggage control upon entering the company;  - testing of personnel who show signs of alcohol or drug use  - prohibiting the access of persons under the influence of alcoholic beverages or drugs  - prohibiting the introduction of alcohol into the organization  - supervision of service providers during the activity;  - limiting access to areas other than those for which the request was made. | - security agent;  - technical manager;  - project manager;  - the food defense team |
| Visitors | Intentional access with:  - toxic and flammable substances;  - prohibited substances (alcohol, drugs, etc.);  -white weapons and sharp objects | 1 | 3 | 3 | medium | Intentional damage / contamination;  Chemical hazard: - spillage of chemical substances - intentional | It can be big - impact on the health of the consumer, impossibility of packaging and delivery, decreased credibility on the market | -prohibition of access with bulky luggage;  - testing of personnel who show signs of alcohol or drug use  - prohibition of access under the influence of alcoholic beverages or drugs  - prohibiting the introduction of alcohol into the organization  - accompanying visitors throughout the visit;  - visitor access limited only to the area for which approval has been received. | - security agent;  - manager of the concerned department  - the food defense team |
| Protection of the product and/or packaging and the company | Access by unauthorized persons | 1 | 3 | 3 | Easy for outside/difficult for inside | Intentional damage / contamination | The difference depends on the type of contamination and the raw material. | Inspection of goods at the reception;  analytical and sensory tests. | security |
|  |  |  |  |  |  |  |  | Products and raw materials are not stored in outdoor areas. | warehouse manager |
|  |  |  |  |  |  |  |  | Any supply vehicle is registered at the control point, its access to the yard is made only after the notification of the responsible department. |  |
| Reception of raw materials/ auxiliary materials | - Physical danger: glass shards, metal chips, sand, cement - intentional | 1 | 3 | 3 | medium | Intentional damage / contamination | It can be big - impact on the health of the consumer, impossibility of packaging and delivery, decreased credibility on the market | - checking the hygiene status of the means of transport; - performing tests;  - checking the integrity of product packaging;  - products whose packaging is damaged are not accepted;  - product quarantine; | -warehouse manager;  -reception committee;  -food defense team |
| Reception of raw materials/ auxiliary materials | -Chemical danger: toxic substances - intentional | 1 | 3 | 3 | medium | Intentional damage / contamination | It can be big - impact on the health of the consumer, impossibility of packaging and delivery, decreased credibility on the market | - checking the hygiene status of the means of transport;  - performing tests;  - checking the integrity of product packaging;  - products whose packaging is damaged are not accepted; - checking the integrity of the seals;  - checking the ingredients recorded on the labels of the delivered products; - product quarantine;  - products whose seal, label, packaging are damaged or which do not correspond to the order are not accepted;  - the access of the supplier/distributor to the warehouse of products/raw materials or to the premises of the factory is prohibited. | -warehouse manager;  -reception committee;  -food defense team. |
| Supply of raw/auxiliary materials | Intentional access of unauthorized personnel in the reception area of raw and auxiliary materials | 1 | 3 | 3 | medium | Intentional damage / contamination | It can be big - impact on the health of the consumer, impossibility of packaging and delivery, decreased credibility on the market | -identifying, registering and notifying the manager.  -selection of suppliers.  - the access of the supplier/distributor inside the factory or any area except for the delivery of raw and auxiliary materials is prohibited. | -security agent  -acquisitions manager  -warewhouse manager;  -reception committee |
| Supply of raw/auxiliary materials | The intentional access of foreigners to society, along with the provision of material goods | 1 | 3 | 3 | medium | Intentional damage / contamination | It can be big - impact on the health of the consumer, impossibility of packaging and delivery, decreased credibility on the market | - control, identification, registration of suppliers/distributors and notification to the manager;  - the prohibition of access to the organization by foreign persons without approval;  - accompanying the supplier/distributor within the organization  - the access of the supplier/distributor inside the factory or any area except for the delivery of raw and auxiliary materials is prohibited | - security guard  - warehouse manage |
| Storage / Delivery / Transport | Physical danger: - the transport of other products together with the finished products in the same machine - on purpose | 2 | 2 | 4 | easy | Intentional damage / contamination | It can be big - impact on the health of the consumer, decrease in credibility on the market | - instruire agenți livratori privind transportul produselor finite; -verificarea periodică pe rute de transport a mijloacelor auto privind respectarea măsurilor stabilite ( igienă, integritate ambalaje, produse transportate); - depozitarea produselor finite în spații special amenajate, controlate d.p.d.v. al igienei și siguranței alimentare, asigurarea integrității | - distribution manager |
| Disinsection, Disinfection, Deratization (DDD) | Chemical danger: substances used in DDD - intentional | 1 | 3 | 3 | medium | Intentional damage / contamination | It can be big - impact on the health of the consumer, impossibility of packaging and delivery, decreased credibility on the market | - station monitoring  -permanent accompaniment of personnel performing DDD flowchart | - designated quality personnel;  - the food defense team. |
